# Health-related quality of life is substantially worse in individuals with plantar heel pain

**DOI:** 10.1038/s41598-022-19588-5

**Published:** 2022-09-19

**Authors:** Karl B. Landorf, Michelle R. Kaminski, Shannon E. Munteanu, Gerard V. Zammit, Hylton B. Menz

**Affiliations:** 1 Discipline of Podiatry, School of Allied Health, Human Services and Sport, La Trobe University, VIC 3086 Australia; 2 La Trobe Sport and Exercise Medicine Research Centre, School of Allied Health, Human Services and Sport, La Trobe University, VIC 3086 Australia; 3 Medical Foot Care, 6/230 Blackshaws Road, Altona North, VIC 3025 Australia

**Keywords:** Musculoskeletal abnormalities, Chronic pain

## Abstract

This study aimed to compare health-related quality of life (HRQoL) in people with and without plantar heel pain (PHP). This was a cross-sectional observational study that compared 50 adult participants with PHP to 25 participants without PHP who were matched for age, sex and body mass index (BMI). HRQoL measures included a generic measure, the Short Form 36 version 2 (SF-36v2), and foot-specific measures, including 100 mm visual analogue scales (VASs) for pain, the Foot Health Status Questionnaire (FHSQ), and the Foot Function Index-Revised (FFI-R). Comparisons in HRQoL between the two groups were conducted using linear regression, with additional adjustment for the comorbidity, osteoarthritis, which was found to be substantially different between the two groups. For generic HRQoL, participants with PHP scored worse in the SF-36v2 physical component summary score (*p* < 0.001, large effect size), but there was no difference in the mental component summary score (*p* = 0.690, very small effect size). Specifically, physical function (*p* < 0.001, very large effect size), role physical (*p* < 0.001, large effect size) and bodily pain (*p* < 0.001, large effect size) in the physical component section were worse in those with PHP. For foot-specific HRQoL, participants with PHP also scored worse in the VASs, the FHSQ and the FFI-R (*p* ≤ 0.005, huge effect sizes for all domains, except FHSQ footwear, which was large effect size, and FFR-R stiffness, activity limitation, and social issues, which were very large effect sizes). After accounting for age, sex, BMI and osteoarthritis, adults with PHP have poorer generic and foot-specific HRQoL.

## Introduction

Plantar heel pain (PHP), also referred to as plantar fasciitis, is a very common foot disorder^1^. Up to 10% of the general adult population are estimated to be affected by PHP^2–8^, but it is also prevalent among younger, athletic populations, such as long distance runners^9–11^ and military personnel^12^. In adults 50 years of age and older who have PHP, the pain is classified as disabling in 82% of those affected^13^. PHP has been found in one study to lead to poorer foot-specific health-related quality of life (HRQoL)^14^, and individuals who experience it have been reported to have increased levels of depression, anxiety and stress^15–17^. Further, PHP has been found to have a prolonged course in many people, with approximately 45% of patients still experiencing pain after 10 years^18^, which leads to a substantial use of health services^19–21^ and a large economic burden^22^.

While many factors have been found to be associated with PHP, the cause of this painful condition is still not fully understood, with studies finding inconsistent results for most of the proposed factors^23–27^. Nevertheless, a recent systematic review found that body mass index (BMI) was consistently associated with PHP, with meta-analysis finding that people with PHP have on average a BMI of 2.28 kg/m^2^ (95% CI 1.34 to 3.22) more than people without PHP^28^. However, increased BMI, overweight and obesity have also been associated with poorer general HRQoL^29–32^, and more specifically, foot-related HRQoL^33^. Therefore, there appears to be a link between obesity, PHP and HRQoL. This is important as not all studies that have investigated HRQoL in PHP have controlled for weight or BMI^14,23,34^. Accordingly, increased weight or BMI may have confounded the findings from some of the studies that have investigated the association of PHP with HRQoL. Therefore, BMI, as well as other important factors such as age and sex, should be accounted for when investigating this issue.

This study aimed to assess differences in HRQoL between those with and without PHP. To achieve this aim, the study compared a group of participants with PHP to a group of participants without PHP that were matched for age, sex and BMI.

## Methods

Some of the methods outlined below have been previously reported in an earlier publication related to this study^35^.

### Study design

This was a cross-sectional observational study and is reported in accordance with the Strengthening the Reporting of Observational Studies in Epidemiology Statement (STROBE)^36^.

### Ethical approval

Ethical approval was obtained from the La Trobe University Human Ethics Committee—Application 14-001—Melbourne, Australia. As part of this approval, the study adhered to the updated Australian National Statement on Ethical Conduct in Human Research (2007)^37^. All participants provided written informed consent prior to recruitment into the study.

### Participants

The participants were 75 community-dwelling adults of either sex from the State of Victoria, Australia. There were two groups of participants: (i) a case group of 50 participants with PHP, and (ii) a control group of 25 matched participants without PHP (i.e. a ratio of 2:1 of cases to controls). Participants in the control group were matched to the case group by age (± 5 years), sex and BMI (± 10%).

### Eligibility criteria

Participants were eligible if they:

(i) Were aged 18 years or over;
(ii) Had PHP for at least 1 month (if recruited to the PHP group);
(iii) Were able to speak basic English, so they could provide informed consent prior to participation, follow instructions during the project, and to answer questions related to the study accurately.

Participants were excluded from the study if they:

(i) Had any conditions (e.g. pregnancy, pacemaker, metal fragments, etc.) that would have precluded them from having the medical imaging related to the over-arching study (the over-arching study was investigating differences in medical imaging findings in people with and without PHP);
(ii) Had any self-reported inflammatory arthritis (e.g. seronegative arthropathy), endocrine/neurological condition (e.g. diabetic peripheral neuropathy, stroke, etc.) or surgery (e.g. amputation, joint fusion, etc.) that had affected lower limb sensation or their ability to walk/run.

### Recruitment

Participants were recruited via several methods including: advertising posters placed at relevant locations (e.g. La Trobe University, private and public health clinics, sporting and senior citizen clubs), the Health Sciences Clinic at La Trobe University, advertisements on relevant web-sites related to health, direct referral from health care practitioners, and via acquaintances of the investigators involved with the study and snowball sampling. Recruitment commenced on 12 January 2015 and was completed on 26 October 2018.

### Sample size

The sample size was one of convenience and was largely dependent on the previously mentioned over-arching study (due to costs associated with the medical imaging). The recruitment ratio of 2 cases (participants with PHP) to 1 control (participant without PHP) was decided on to minimise the burden of recruiting age-, sex- and BMI-matched control participants.

### Setting

The study was performed in one of three settings: (i) a research room in the Health Sciences Clinic at La Trobe University, (ii) a health science clinical tutorial room at La Trobe University, or (iii) in a room at a participant’s home with a hard floor (e.g. linoleum, concrete or wood).

### Protocol

Data were collected by one of three of the authors (KBL, MRK, GVZ), all of whom were registered podiatrists with more than 10 years of experience at the time of data collection. Following informed consent, participants were examined in one session that took approximately one to one-and-a-half hours. Data were collected using a standardised assessment form.

### Data collected

#### General participant information

Participants were asked for general participant information such as date of birth (which allowed calculation of age), sex, and general medical history (including co-morbidities such as diabetes, hypertension and osteoarthritis). In addition, if they were a participant with PHP, they were also asked for the duration of their symptoms.

#### Physical characteristics

Participants had their height (in metres) and weight (in kilograms) measured; subsequently, their BMI was calculated in kg/m^2^. The normal range for BMI as recommended by the World Health Organization (WHO) is 18.5 to 24.9 kg/m^2^, overweight is 25.0 to 29.9 kg/m^2^ and obese is ≥ 30.0 kg/m^2^^38^. In addition, their waist and hip circumference was measured (in cm); subsequently their waist-hip ratio calculated. The WHO recommend that if the waist-hip ratio is ≥ 0.90 for men and ≥ 0.85 for women then this indicates abdominal obesity, which indicates a substantially increase risk of metabolic complications^39^.

#### Medications

Participants were asked to list any prescribed medications that they were currently using.

#### Level of education

Participants were asked to report their highest level of education that they had completed, which was categorised as: (i) no formal, (ii) less than primary school, (iii) primary school completed, (iv) high school (or equivalent) completed, (v) TAFE (technical college) completed, (vi) college/university completed, (vii) post graduate degree completed, (viii) don’t know, (ix) other (please state).

#### Physical activity

Physical activity was measured by the Stanford Activity Questionnaire, which was expressed as kilocalories expended per day^40,41^.

#### Generic and foot-specific HRQoL

In the absence of clear definitions of HRQoL^42^, we chose to include patient-reported health status measures that empirically evaluate the way health affects QoL^43^. We also included visual analogue scales in this study as they are commonly used in clinical practice and clinically-based research to determine pain levels at different timepoints and with different activities, although we recognise that some researchers might argue that the isolated measurement of pain is not technically a HRQoL measure on its own. The suite of measures we used are detailed below.

Generic HRQoL was measured with the Short Form-36™ Standard Health Survey, US Version 2 (SF-36), QualityMetric Incorporated and Medical Outcomes Trust^44–46^, and domains included physical function, role physical (role limitations due to physical health problems), bodily pain, general health, which made up the physical component, and vitality, social functioning, role emotional (role limitations due to emotional problems), mental health, which made up the mental component. Scores for the SF-36 range from 0 to 100 with lower scores indicating poorer health status and higher scores indicating better health status.

Foot -specific HRQoL was measured using the 100 mm Visual Analogue Scale (VAS)^47^, Foot Health Status Questionnaire (FHSQ)^48^, and the Foot Function Index-Revised (FFI-R)^49,50^. Three 100 mm VAS assessments were taken that covered the typical course of PHP over the last week: first step pain (heel pain when first arising out of bed), average heel pain for today, and average heel pain for the last week. A score of 0 on a VAS represents no pain and a score of 100 represents the worst pain imaginable. For the FHSQ, domains include foot pain, foot function, footwear and general foot health. Scores for the FHSQ range from 0 to 100 with lower scores indicating poorer foot health status and higher scores indicating better foot health status for an individual. For the FFI-R, sub-scales include (foot) pain, (foot) stiffness, difficulty, activity limitation, and an overall FFI-R score can also be calculated. Scores for the FFI-R range from 0 to 100 with lower scores indicating better foot health status and higher scores indicating poorer foot health status (note: this scoring is opposite to the FHSQ and SF-36).

### Data analysis

Data were analysed using IBM Statistical Package for the Social Sciences (SPSS) Version 26.0 (IBM Corporation, Armonk, NY). All continuous data were first explored for normality prior to inferential analysis. For categorical data, chi-square tests were used to test for differences between groups. For ordinal data, or non-normally distributed continuous data, medians and inter-quartile ranges (IQRs) were calculated and Mann–Whitney *U* tests were used to test for differences between groups. For continuous data, mean differences with 95% confidence intervals were calculated and unpaired *t*-tests were used to test for differences between groups. If any comorbidities were found to be substantially different between groups when evaluating HRQoL data, we conducted linear regression analyses to adjust for this covariate, so its influence on the findings was controlled for. The critical level of significance was set at *α* = 0.05. Beta coefficients were calculated for group membership (i.e. with or without PHP) and the covariate to provide an indication of the relative importance of these factors in explaining variance in HRQoL, and adjusted *R* square values were calculated for each model. To provide an estimation of the size of the differences in HRQoL between the groups, Cohen’s *d* effect size values were calculated for HRQoL measures by dividing the mean difference between the groups by the average of the standard deviations for both groups. Interpretations of the calculated effect sizes were taken from Sawilowsky^51^, which are 0.01 = very small, 0.2 = small, 0.5 = medium, 0.8 = large, 1.2 = very large, and 2.0 = huge.

## Results

### Participant characteristics

Seventy-five participants were recruited into the study. There were 50 participants with PHP (case group) and 25 participants without PHP (control group) that were matched for age, sex and BMI. There were no statistically significant differences in age, sex or BMI between the two groups (Table 1). The mean age was 49.1 years in the PHP group and 48.9 years in the control group, with a range for all participants of 23 to 75 years. Women comprised 58% of the PHP group and 56% of the control group. The mean BMI was 30.6 kg/m^2^ in the PHP group and 30.2 kg/m^2^ in the control group, which indicates participants were, on average, obese; although, the range of BMIs for all participants was from normal to very severely obese (range 20.1 to 47.7 kg/m^2^). In addition to BMI, the measure of abdominal obesity (via the waist-hip ratio) was also well matched between the PHP and the control groups two groups, as were education level, number of self-reported medications, and activity levels. Regarding symptoms in the PHP group, the median duration of symptoms was 6.5 months with a range of 1.0 to 80.0 months. Their mean first step pain on a 100 mm VAS was 53 mm, mean pain on the day of their assessment was 39 mm, and their mean pain in the last 7 days was 50 mm.

**Table 1:** Participant characteristics—values are means (SDs) unless otherwise stated.

Variable	With PHP (*n* = 50)	Without PHP (*n* = 25)	Mean difference (95% CI)	*P*-value
Age (years)	49.1 (11.6)	48.9 (9.9)	0.2 (− 5.2, 5.6)	0.947
Sex—no. of females (%)	29 (58%)	14 (56%)	*N/A*	0.869^a^
Height (m)	1.68 (0.10)	1.73 (0.12)	− 0.05 (− 0.11, 0.00)	0.051
Weight (kg)	86.1 (17.5)	90.3 (21.4)	− 4.2 (− 13.4, 5.0)	0.370
BMI (kg/m^2^)	30.6 (6.2)	30.2 (7.2)	0.4 (− 2.8, 3.6)	0.813
Waist circumference (cm)	100.9 (11.4)	101.7 (19.4)	− 0.8 (− 9.3, 7.8)^b^	0.858^b^
Hip circumference (cm)	112.0 (12.6)	111.1 (15.3)	0.9 (− 5.7, 7.5)	0.784
Waist-hip ratio	0.90 (0.06)	0.91 (0.09)	− 0.01 (− 0.05, 0.03)	0.614
**Duration of symptoms**				
Median (IQR)^c^	6.5 (3.0–12.0)	0.0 (0.0–0.0)	*N/A*	*N/A*
**Education level—category**				
Median (IQR)	6 (4–7)	6 (5–6.5)	*N/A*	0.785^d^
**No. of prescribed medications**				
Median (IQR)^c^	0 (0–1)	0 (0–1)	*N/A*	0.430^d^
Activity level (kilocalories expended per day)	3,745 (1,012)	3,689 (1,034)	56 (− 441, 554)	0.823

*N/A* = Not applicable.

^a^*P*-value relates to Chi-squared test.

^b^Mean difference, 95% CIs and *p*-value adjusted as Levene’s Test for Equality of Variances was significant (*p* < 0.05).

^c^Median (IQR) reported as variable not normally distributed.

^d^*P*-value relates to Mann–Whitney *U* test.

Groups were also compared for the prevalence of comorbidities that may have affected HRQoL. There were no statistically significant differences between groups (Table 2). However, the prevalence of the comorbidity osteoarthritis was substantially different between the groups (38% versus 16%), even though it was not statistically significant (*p* = 0.051), so we elected to adjust for this in the HRQoL comparisons.

**Table 2:** Prevalence of comorbidities in the groups with and without PHP.

Variable	With PHP (*n* = 50)	Without PHP (*n* = 25)	*P*-value^a^
Diabetes^b^	1 (2%)	1 (4%)	0.612
Hypertension	5 (10%)	2 (8%)	0.779
Heart disease	1 (2%)	1 (4%)	0.612
Hormone replacement therapy	1 (2%)	1 (4%)	0.612
Hypercholesterolaemia	2 (4%)	3 (12%)	0.190
Thyroid disease	3 (6%)	1 (4%)	0.716
Osteoarthritis	19 (38%)	4 (16%)	0.051
Rheumatoid/inflammatory arthritis^c^	1 (2%)^c^	0 (0%)	0.477
Peripheral vascular disease	0 (0%)	0 (0%)	*N/A*
Other foot conditions leading to foot surgery^d^	4 (8%)	3 (12%)	0.575
Other conditions^e^	6 (12%)	7 (28%)	0.084

Data provided for groups are number (percentage).

*N/A* = Not applicable—no cases in either group.

^a^*P*-value relates to Chi-squared test.

^b^Cases did not have any related condition (e.g. diabetic peripheral neuropathy) that affected lower limb sensation or their ability to walk/run.

^c^Case relates to a previous attack of gout—participant had no symptoms or substantial bone abnormality.

^d^Conditions included: calcaneo-cuboid surgery, Achilles tendon surgery, peroneal tendon surgery, Morton’s neuroma surgery, and fractured ankle requiring surgery. Conditions were assessed as to not warrant exclusion.

^e^Conditions included: cataracts, rotator cuff issue, Baker’s cyst, previous anterior cruciate ligament and meniscus surgery, previous total knee replacement, previous deep vein thrombosis, patella-femoral joint pain, nerve pain not specific to feet, HIV (human immunodeficiency virus) infection, verruca on plantar feet, depression, hiatus hernia/gastro-oesophageal reflux disease, psoriasis, eczema, coeliac disease, treatment for alcohol dependence. Conditions were assessed as to not warrant exclusion.

### Generic health-related quality of life

There were significant differences between the PHP and control groups in generic HRQoL; namely the SF-36—Table 3. Overall, the physical health component score was significantly lower in the PHP group, but there was no difference in the mental health component score. When individual domains were compared in the physical health component of the SF-36, the physical function, role physical and bodily pain domain scores were significantly lower in the PHP group, but there was no difference between the groups for the general health domain. When individual domains were compared in the mental health component, there were no significant differences between groups. Effect sizes ranged from large to very large for domains in the physical health component that were found to be statistically significant.

**Table 3:** Comparison of generic health-related quality of life as measured with the SF-36 in participants with and without PHP—values are means (SDs).

Component	Domain	With PHP (*n* = 50)	Without PHP (*n* = 25)	Adjusted mean difference (95% CI)^a^	Adjusted *R* square	Standardised *β* coefficients^b^	*P*-value	Cohen’s *d* effect size (interpretation)^c^
Physical	Overall	42.9 (7.7)	51.7 (8.7)	− 7.9 (− 11.9, − 4.0)	0.229	− 0.418, − 0.197	< 0.001	1.11 (large)
	Physical function	68.6 (20.3)	89.8 (11.9)	− 19.0 (− 27.8, − 10.2)	0.271	− 0.440, − 0.228	< 0.001	1.20 (very large)
	Role physical	66.4 (26.6)	89.8 (21.6)	− 21.0 (− 33.4, − 8.6)	0.175	− 0.365, − 0.184	< 0.001	0.95 (large)
	Bodily pain	57.8 (21.6)	77.0 (16.9)	− 17.9 (− 28.0, − 7.8)	0.162	− 0.386, − 0.121	< 0.001	0.96 (large)
	General health	71.7 (15.8)	73.7 (16.2)	− 1.2 (− 9.1, 6.8)	− 0.014	− 0.035, − 0.103	0.769	0.13 (very small)
Mental	Overall	53.7 (7.9)	53.3 (6.6)	0.8 (− 3.0, 4.5)	− 0.020	0.048, − 0.085	0.690	0.05 (very small)
	Vitality	58.5 (17.7)	60.3 (18.3)	0.3 (− 8.5, 9.1)	0.031	0.008, − 0.241	0.949	0.10 (very small)
	Social functioning	83.0 (19.8)	91.5 (11.8)	− 8.1 (− 16.9, 0.8)	0.027	− 0.213, − 0.052	0.075	0.49 (small)
	Role emotional	87.0 (20.2)	96.3 (6.4)	− 6.8 (− 15.0, 1.3)	0.128	− 0.186, − 0.302	0.099	0.56 (medium)
	Mental health	79.4 (13.4)	81.0 (12.3)	− 2.0 (− 8.6, 4.6)	− 0.020	− 0.073, 0.064	0.548	0.12 (very small)

Scores for the SF-36 range from 0 to 100 with lower scores indicating poorer health status and higher scores indicating better health status for an individual.

^a^Mean difference adjusted for the covariate osteoarthritis.

^b^Two standardised *β* coefficients are provided—the first for group (i.e. with or without PHP) and the second for the covariate (i.e. comorbidity) osteoarthritis.

^c^Interpretations for the Cohen’s *d* effect sizes were taken from Sawilowsky ^49^.

### Foot-specific health-related quality of life

There were also significant differences between the PHP and control groups in foot-specific HRQoL; namely VAS pain (first step pain, average pain and average pain in the last 7 days), the FHSQ (all domains), and the FFI-R (all sub-scales)—Table 4. For the PHP group, all three timepoints for the VAS pain and all domains for the FHSQ and FFI-R (including the social issues domain) scored worse. Effect sizes ranged from very large to huge.

**Table 4:** Comparison of foot-specific health-related quality of life in participants with and without PHP—values are means (SDs).

Measure	Domain/sub-scale	With PHP (*n* = 50)	Without PHP (*n* = 25)	Adjusted mean difference (95% CI)^a^	Adjusted *R* square	*β* coefficients^b^	*P*-value	Cohen’s *d* effect size (interpretation)^c^
100 mm VAS	First step pain	53.3 (26.5)	0.0 (0.0)	53.2 (42.2, 64.2)	0.567	0.760, 0.004	< 0.001	2.49 (huge)
	Average pain today	39.7 (20.7)	0.0 (0.0)	39.6 (31.1, 48.2)	0.542	0.744, 0.006	< 0.001	2.37 (huge)
	Average pain last 7 days	50.8 (24.5)	0.0 (0.0)	49.7 (39.6, 59.8)	0.587	0.754, 0.072	< 0.001	2.57 (huge)
FHSQ	Pain	40.3 (20.4)	93.8 (10.8)	− 53.1 (− 62.1, − 44.1)	0.664	− 0.815, − 0.022	< 0.001	3.04 (huge)
	Function	60.4 (21.2)	97.3 (7.0)	− 35.0 (− 43.8, − 26.2)	0.505	− 0.667, − 0.160	< 0.001	2.10 (huge)
	Footwear	52.7 (27.0)	74.7 (29.0)	− 19.8 (− 33.6, − 6.0)	0.125	− 0.320, − 0.156	0.005	0.81 (large)
	General foot health	37.4 (28.6)	87.8 (15.5)	− 49.9 (− 62.6, − 37.3)	0.467	− 0.686, − 0.032	< 0.001	2.04 (huge)
FFI-R	Pain	65.2 (12.4)	26.2 (3.0)	39.1 (33.9, 44.3)	0.760	0.878, − 0.013	< 0.001	3.84 (huge)
	Stiffness	50.9 (20.6)	26.9 (4.3)	24.7 (16.1, 33.3)	0.297	0.573, − 0.064	< 0.001	1.43 (very large)
	Difficulty	53.3 (14.6)	26.4 (4.6)	26.0 (19.8, 32.1)	0.523	0.697, 0.117	< 0.001	2.23 (huge)
	Activity limitation	36.7 (9.6)	25.6 (2.9)	11.1 (7.0, 15.1)	0.281	0.548, 0.004	< 0.001	1.40 (very large)
	Social issues	40.4 (12.8)	26.8 (4.0)	12.6 (7.3, 17.9)	0.280	0.479, 0.179	< 0.001	1.28 (very large)
	Overall FFI	49.3 (9.8)	26.4 (3.3)	22.7 (18.5, 26.8)	0.630	0.790, 0.040	< 0.001	2.81 (huge)

For the *VAS*, a score of 0 represents no pain and a score of 100 represents the worst pain imaginable. For the *FHSQ*, scores range from 0 to 100 with lower scores indicating poorer foot health status and higher scores indicating better foot health status for an individual. For the *FFI-R*, scores range from 0 to 100 with lower scores indicating better foot health status and higher scores indicating poorer health status for an individual (note: this scoring is opposite to the *FHSQ* and *SF-36*).

*VAS* Visual Analogue Scale, *FHSQ* Foot Health Status Questionnaire, *FFI-R* Foot Function Index—Revised.

^a^ Mean difference adjusted for the covariate osteoarthritis.

^b^ Two standardised *β* coefficients are provided—the first for group (i.e. with or without PHP) and the second for the covariate (i.e. comorbidity) osteoarthritis.

^c^ Interpretations for the Cohen’s *d* effect sizes were taken from Sawilowsky^49^.

## Discussion

Our study aimed to assess differences in HRQoL between adults with and without PHP while accounting for body mass. To achieve the aim of the study we compared a group of participants with PHP to a group of participants without PHP who were matched for BMI, as well as age and sex. Participants in the two groups were well matched on these three key matching criteria. Importantly, participants were well matched for BMI, a critical variable in the context of the aim of our study. In addition, the PHP and control groups also had similar abdominal obesity (via the waist-hip ratio), education levels, number of self-reported prescribed medications being taken, and activity levels. However, one comorbidity, osteoarthritis, was found to be substantially more prevalent in the PHP group, so we elected to adjust for this. With this approach, we believe that potential confounding was minimised, so we subsequently progressed to compare the two groups for generic and foot-specific HRQoL.

Participants with PHP were found to have worse generic HRQoL scores as assessed with the SF-36, however this was isolated to only domains in the role physical component. The overall physical component score, and the individual domains of physical function, role physical and bodily pain were found to be substantially worse in PHP participants with effect sizes interpreted as large to very large. We did not find any substantial differences in the mental component of the SF-36, which is in contrast to the findings of a recent systematic review^17^ that found moderate level evidence for associations between psychosocial variables and PHP. However, the studies in this systematic review used different methodologies, and most did not specifically account for BMI and other comorbidities like we did. Accordingly, previous research that has found such associations may have been confounded by comorbidities. The SF-36 scores observed in participants without PHP in our study are also similar to population norms from the 1995 National Health Survey conducted by the Australian Bureau of Statistics^52^. Therefore, although this survey was conducted more than 2 decades ago, it shows that our control group were largely representative of the population from the perspective of generic HRQoL, so participants with PHP in our study had substantially worse generic physical HRQoL than population norms. This provides a benchmark for the impact of PHP on individuals who experience it.

Participants with PHP were also found to have substantially worse foot-specific HRQoL, as assessed with the three foot-specific measures that we used: the VAS, the FHSQ, and the FFI-R. Furthermore, all domains/sub-scales of these outcome measures were found to be substantially worse in participants with PHP.

Firstly, three different VASs for pain were assessed that covered the typical course of PHP over the last week (first step pain, average pain today, and average pain in the last 7 days), all of which were found to be substantially worse in the PHP group with effect sizes interpreted as huge for all three. The issue of first step pain (heel pain when first arising out of bed) is characteristic of PHP^1^, as is daily pain that worsens with activity and more chronic pain that can last many years in some individuals^18^.

Secondly, all four domains of the FHSQ (pain, function, footwear and general foot health) were found to be substantially worse in the PHP group with effect sizes interpreted as huge, except for footwear, which was large. These findings are also greater than those found to be clinically meaningful^53,54^. A previous study by Irving et al.^14^ that adjusted for differences in BMI between a PHP and a control group also found individuals with PHP scored substantially worse in all four domains. The pain experienced with PHP is located on the plantar aspect of the heel and because standing and walking (and for some, running) are key activities for daily living, the findings from our study and the study conducted by Irving et al. are not surprising. There is one other study by Palomo-Lopez and co-workers^34^ that also assessed foot-specific HRQoL in adults with PHP, but their aim was to compare males and females with PHP, rather than adults with PHP in general to a control group without PHP. They found that females had worse foot-specific HRQoL in the FHSQ domains of foot function and footwear (but not pain and general foot health). The issue of footwear difficulties in people with PHP has not been extensively investigated using robust study designs, aside from a study by Sullivan et al.^55^, which our findings agree with. We believe, therefore, that future studies investigating footwear in PHP are warranted, including evaluations of the effectiveness of different types of footwear in appropriately powered randomised trials.

Thirdly, all domains or sub-scales of the FFI-R were found to be substantially worse in the PHP group with effect sizes interpreted as very large, except for pain and difficulty, which were huge. The FFI-R is similar to the FHSQ in that it empirically assesses foot health and its impact on QoL, although it does contain different domains or sub-scales, which are pain, stiffness, difficulty, activity limitation, social issues, and an overall FFI-R score can also be calculated. While overall, the FFI-R’s pain, stiffness, difficulty and activity limitation sub-scales are likely to measure similar issues to the FHSQ’s pain and function domains, albeit under four domains not two, the social issues sub-scale is unique to the FFI-R. The finding of a worse social issues score for the PHP group is in contrast to our findings for the generic HRQoL measure, the SF-36, discussed previously. These contrasting findings may simply be due to differences in foot specific versus generic HRQoL measures (i.e. foot specific is more sensitive to detect differences), but it may also be due to the sample size for our study not being large enough—as it was limited to the over-arching study—to detect clinically meaningful differences in generic HRQoL. Accordingly, we believe that further research is warranted on PHP’s effect on an individual’s psychosocial health (e.g. health concerns, mental health, ability to carry out daily activities and to engage socially)^49^.

In summary, adults with PHP have poorer generic and foot-specific HRQoL. The pain associated with PHP leads to affected individuals not only having poorer foot and broader physical function, but also poorer social functioning as detected by the FFI-R. The pain experienced under the heel, and the wider functional limitations that occur because of this pain, are hallmarks of PHP and there are many conservative treatments, such as stretching^56^, foot taping^56^, foot orthoses^57^, extracorporeal shockwave therapy^58^, and corticosteroid injection^59^ that are targeted at these symptoms and impairments. However, the broader psychosocial issues, including its impact on social functioning and roles an individual participates in are less well understood and deserve greater investigation^17^. While there is some early evidence that psychosocial factors, such as anxiety and depression, are factors in PHP^15,16,60^, the specific finding of poorer social functioning has only recently been reported in one qualitative study in which 18 participants underwent a semi-structured interview^61^. In addition, only one study has investigated PHP among assembly plant workers^25^, but this focused on risk factors, so further investigation of role limitations in work and other activities is warranted to better understand the broader economic and societal burdens of this condition.

There are several strengths to our study that should be highlighted. Our study matched a general sample of adult participants using a broad recruitment strategy with and without PHP for BMI. This is important as BMI has consistently been associated with PHP^28,62^, so unless controlled for, BMI may be a confounding factor in observational studies. We also adjusted for osteoarthritis, which after recruitment was found to be more prevalent in the PHP group. In addition, we utilised valid and reliable patient-reported outcome measures to assess HRQoL. Our findings, therefore, should be generalisable to the wider population of adults with PHP. Participants with PHP in our study were, on average, in their late 40s, although there was a substantial age range (mean 49 years, range of 23 to 75 years). The majority of participants (58%) with PHP were women, and they were on average obese (mean BMI was 30.6 kg/m^2^ with a range of 20.1 to 47.7 kg/m^2^). These values are consistent with other studies from different countries, including epidemiological investigations^7,8,63^, investigations of risk factors^23,64–66^, and randomised trials^56,58^. Regarding symptoms in the PHP group, the median duration of symptoms was 6.5 months in the PHP group with a range of 1 to 80 months. Their mean first step pain was 53 mm (measured on a 100 mm VAS), mean pain on the day of their assessment was 39 mm, and their mean pain in the last 7 days was 50 mm, which equates to pain levels that are moderate. Considered together, these findings indicate that the participants in this study are generalisable to the broader population of people with PHP, particularly middle-aged individuals, who make up the majority of cases^7,63^. Our study is also the first to include multiple measures of the impact of PHP on HRQoL, which makes it unique in that their findings support each other. The HRQoL measures we used included both generic and foot-specific measures, as well as multiple foot specific measures.

Our study has four limitations that need to be considered. Firstly, the sample size for the study was one of convenience as it was dictated by the limits of the over-arching study. Some of our findings, therefore, may be limited by sub‐optimal precision in the estimates of difference between groups. Secondly, we chose to recruit participants from the wider adult population with PHP, so our results are generalisable to these people, not to specific sub-populations with PHP (e.g. younger, active adults, such as long distance runners). Thirdly, our study did not utilise blinded investigators, however because the HRQoL measures were self-reported (i.e. patient-reported outcome measures), we do not believe that assessor bias was an issue. Finally, our study was cross-sectional, so we cannot make inferences about cause and effect.

## Conclusion

After accounting for age, sex, body mass and osteoarthritis, adults with PHP have poorer generic and foot-specific HRQoL. While pain and functional impairment associated with PHP have already received considerable investigation, further research is needed to fully understand its impact on mental health, specifically its effects on the ability for an individual to function socially and on the roles that they would normally participate in, including work.

## Data Availability

The datasets generated during and/or analysed during this study are available from the corresponding author on reasonable request.
